# Expression of recombinant HBD3 protein that reduces *Mycobacterial* infection capacity

**DOI:** 10.1186/s13568-018-0573-8

**Published:** 2018-03-20

**Authors:** Feng Su, Xin Chen, Xin Liu, Guanghui Liu, Yong Zhang

**Affiliations:** 1 College of Animal Science and Veterinary Medicine, Shandong Agricultural University, Taian, Shandong China; 2 College of Veterinary Medicine, Northwest A&F University, Yangling, Shaanxi China

**Keywords:** HBD3, *M. bovis*, Prokaryotic expression, Infection capacity

## Abstract

Bovine tuberculosis is a disease caused by *Mycobacterium bovis* (*M. bovis*) that leads to great economic losses in cattle production. The discovery of a reasonable bioagent to reduce *M. bovis* infection risk and environment contamination becomes significant and urgent. Previous study reported that human β-defensin-3 (HBD3) participated in *Mycobacterial* immunity and was recognized as a suitable candidate reagent. However, its minimal inhibitory concentration to *M. bovis* is not yet reported. In this study, we first purified HBD3 protein by recombinant-DNA technology and prokaryotic expression system. Subsequently, anti-bacterial tests were used to evaluate the basic bioactivity of the protein. Results revealed that recombinant HBD3 (rHBD3) protein inhibits *Staphylococcus* multiplication but not the host *Escherichia coli*. The growth curve of *M. bovis* showed that rHBD3 protein controls the proliferation of *M. bovis* in 20 μg/ml concentration. In addition, rHBD3 protein-incubated *M. bovis* exhibited reduced infectivity to alveolar epithelial cells and macrophages. In conclusion, the expression of rHBD3 protein is a potential ideal bio-regent for reducing *M. bovis* infection.

## Introduction

Bovine tuberculosis is a chronic disease caused by *Mycobacterium bovis* (*M. bovis*) and is mainly characterized by the formation of granulomas in the lung and other organs (Muller et al. 2013). The disease causes great economic losses in cattle production every year. Killing of the suffering cattle as a common approach to reduce *M. bovis* prevalence results in great economic losses (Su et al. 2016). Our understanding on reducing these losses mainly focuses on tuberculosis prevention. This approach is divided into two major approaches. One of which is the vaccine method; however, the inefficient protection ability and potential virulence risk of antigen limits its application in cattle (Buddle et al. 2011). The other one is the prophylactic application of antibiotics in rational dosage. However, the ecological damage of probiotics in the animal body, and the pathogenic bacteria variation in the environment increased the cattle illness occurrence risk (Allen et al. 2010; Martinez 2008, 2009). Thus, a reasonable bio-agent is needed to reduce bovine *Mycobacterial* infection risk and environment contamination. Small potential human defensins that participated in the *Mycobacterial* immunity were recognized as suitable candidate agents that can reduce this risk (Driss et al. 2009; Rivas-Santiago et al. 2006). Among these defensins, human β-defensin 3 (HBD3) is an ideal potential one as previously reported.

As a star protein of β defensins that acts both as an antimicrobial agent and chemo-attractant molecule, HBD3 has an effective antibacterial activity for many different bacteria (Hoover et al. 2003; Maisetta et al. 2003). Moreover, the HBD3 protein exhibited low red cytotoxicity in high salt concentration relative to other proteins (Quinones-Mateu et al. 2003; Sun et al. 2005). High concentrated HBD3 protein appears in early *M. bovis* infection period and is reduced in the latent stage. HBD3 protein participates in *Mycobacterial* clearance and is also associated with long-term control of *Mycobacterial* proliferation (Rivas-Santiago et al. 2006). Previous study reported that His-HBD3 recombinant protein exhibited anti-*Mycobacterial* capacity to H37Rv strain (Corrales-Garcia et al. 2013). A recent study also confirmed that the expression of HBD3 protein in cattle evidently reduced the susceptibility to *M. bovis* infection (Su et al. 2016). However, the accurate inhibition concentration of this purified peptide (without His-tag) has not been determined yet.

The expression of HBD3 protein in vivo solely relies on an EGFR/MAPK/AP-1 dependent pathway, and the HBD3 protein production mainly depends on the pathogenic bacteria intensity (Steubesand et al. 2009). Hence, HBD3 protein maintaining a natural physiological concentration which is not efficiently to resist the high-density bacteria invasion within a short time. Prokaryotic expression of HBD3 fusion protein was initially designed and optimized by Huang et al. (2006, 2007). However, the existence of His tag reduced the production of HBD3. Additionally, N-terminal electric variation caused by His tag affected its biological characteristics (Hoover et al. 2003). Thus, the obtained soluble rHBD3 and its anti-*M. bovis* capacity analysis are critical for the tuberculosis control in cow.

In this study, highly efficient soluble rHBD3 protein was expressed by GST prokaryotic expression system. The anti-bacterial ability and anti-*M. bovis* capacity of rHBD3 protein were evaluated.

## Materials and methods

### Expression vector construction

The pGEX-5X-1 vector was purchased in Amersham Pharmacia Biotech (Piscataway, New Jersey, USA) and was then amplified in LB culture medium (tryptone 1%, yeast extract 0.5%, NaCl 1%, pH: 7.0). HBD3 sequence was obtained by mature protein sequence, and optimal sequence was synthesized in Invitrogen Company (Invitrogen, Carlsbad, CA). The optimal designed sequences are as follows: (GTGATCATTAACACTCTGCAAAAATATTACTGCCGCGTGCGTGGTGGCCGTTGTGCGGTTCTGTCCTGTCTGCCGAAAGAAGAGCAGATCGGCAAATGCTCTACCCGCGGTCGTAAATGCTGCCGTCGTAAAAAGTAATGATGAGAATTC). The vector and optimal sequence were double digested by *Bam*HI, *Eco*RI and *Bcl*I, *Eco*RI separately and were connected by T4 ligase enzyme. Recombinant of pGEX-5X-HBD3 plasmid was identified by digestion and sequencing.

### Determination of optimal induced conditions

Optimal-induced condition test was operated to obtain more soluble HBD3 protein. In the previous study, the optimal-induced temperature and IPTG concentration were tested. Hence, we selected 28 °C and 1 mM IPTG as optimized conditions, and the optimal inducement time was evaluated. Bacteria were collected at 3, 5, 7, and 9 h, and then the bacteria were cracked by lysozyme. Supernatant and sediment were separately collected after being centrifuged on 4000 rpm for 5 min. Optimal induce time was analyzed by SDS-PAGE.

### Purification of GST-HBD3 fusion protein

The *E. coli* bacteria that containing pEGX-5X-HBD3 plasmid was amplified in LB medium (0.5% yeast extract, 1% tryptone, 1% NaCl) containing 50 μg ampicillin/ml. IPTG was then added into the medium when its OD (600) reached 0.4. The bacteria were harvested and collected by centrifuging at 4000 rpm for 20 min. The purified GST-HBD3 recombinant protein was obtained as previously reported (Huang et al. 2007).

### Identification and harvest of rHBD3 protein

Purified HBD3 recombinant protein was harvested by cleavage of GST-HBD3 protein using *Factor Xa* (NEB) at room temperature. The enzyme and Xa buffer (20 mM Tris–HCl: pH 8.0 with 100 mM NaCl and 2 mM CaCl_2_) were incorporated into the GST-HBD3 protein at 23 °C for 6 h, and the total digested protein was dialyzed by binding buffer (50 mM NaH_2_PO_4_–Na_2_HPO_4_, 1 M NaCl, pH 7.4). The purified protein was obtained after flowing through Sepharose^®^ Fast Flow system. The protein was checked by Tris-tricine-SDS-PAGE and was confirmed by western blot analysis. After being transferred on a PVDF membrane, the protein was confirmed after incubation with HBD3 antibody (Sigma, St. Louis, MO) at 4 °C overnight and with goat anti rabbit IgG (Sigma, St. Louis, MO) for 2 h.

### Antimicrobial activity of purified rHBD3 on BL21 (DE3) *E. coli* and *Staphylococcus*

Antimicrobial activity tests of HBD3 protein were evaluated for its biological function by a bacteria growth curve test for BL21 host bacteria and *Staphylococcus aureus* (ATCC25923). Exponentially growing bacteria were re-suspended in 10 mM sodium phosphate buffer (pH 7.4) to reach a density of 5 × 10^7^ CFU/ml. Ten microliters of each bacterial suspension was exposed for 1.5 h under the appropriate culture condition to different treatments in 100 μl of 10 mM sodium phosphate buffer (pH 7.4). The number of bacteria as directed by the optical density (OD 600) was measured every 30 min. The inhibition zone tests were performed to identify its inhibitory concentration.

### Determination of minimal inhibition concentration (MIC) on *M. bovis*

*Mycobacteria bovis* virulent strain C68014 were purchased from the China Institute of Veterinary Drugs Control (Beijing, China) and cultured on Middlebrook 7H10 medium (Difco Laboratories, Detroit, MI) for 20 days. The colonies were then transferred to Middlebrook 7H9 modified medium (Difco Laboratories, Detroit, MI, USA) for 20 days. Determination of MIC was performed in *M. bovis* growth curve. Serial concentrated peptide dilutions in Middle Brook 7H9 broth were prepared. Subsequently, 50 μl of this suspension was mixed with 50 μl of the peptide dilution in each well. After incubation for 12, 24, 48, 60, 72, 84, and 96 h at 37 °C, the bacterial number directed by OD600 data was measured.

### In vitro infection of A549 cells

Human type II alveolar pneumocytes A549 (CCL185) and RAW 264.7 cells were separately cultured in 75 cm^2^ culture flasks (CAS, Shanghai, China) with antibiotic-free Dulbecco’s modified Eagle’s medium (HyClone laboratories, Logan, Utah) supplemented with 10% fetal calf serum (Gibco BRL, Grand Island, NY). A549 and RAW 264.7 cells were pre-incubated in the medium for 24 h prior to *M. bovis* infection. The cells were infected with *M. bovis* at MOI 10:1 for 24 h. *M. bovis* was then detected by Auramine O (Sigma) methods according to the manufacturer’s instructions.

### Cell apoptosis detection

*Mycobacteria bovis* (MOI: 10:1) and HBD3 protein were separately co-incubated with A549 and RAW 264.7 cells for 24 h. Cell apoptosis was then evaluated by DeadEnd TM Fluorometric Tunel System (Promega Corporation, Madison, WI, USA) and cell nucleus dying by PI according to the instruction book (Sigma Co, St. Louis, MO). Cell apoptosis ratio was then detected by flow cytometry.

### Statistical analysis

Inhibited concentration, FCM and CFU test results were analyzed using SPASS software (SPSS, Chicago, IL, USA). The changes are presented as mean ± SEM and were compared using one-way ANOVA followed by Newman–Keuls test. P values < 0.05 were considered as statistically significant.

## Results

### Construction of pGEX-5X-HBD3 vector

**Fig. 1:**
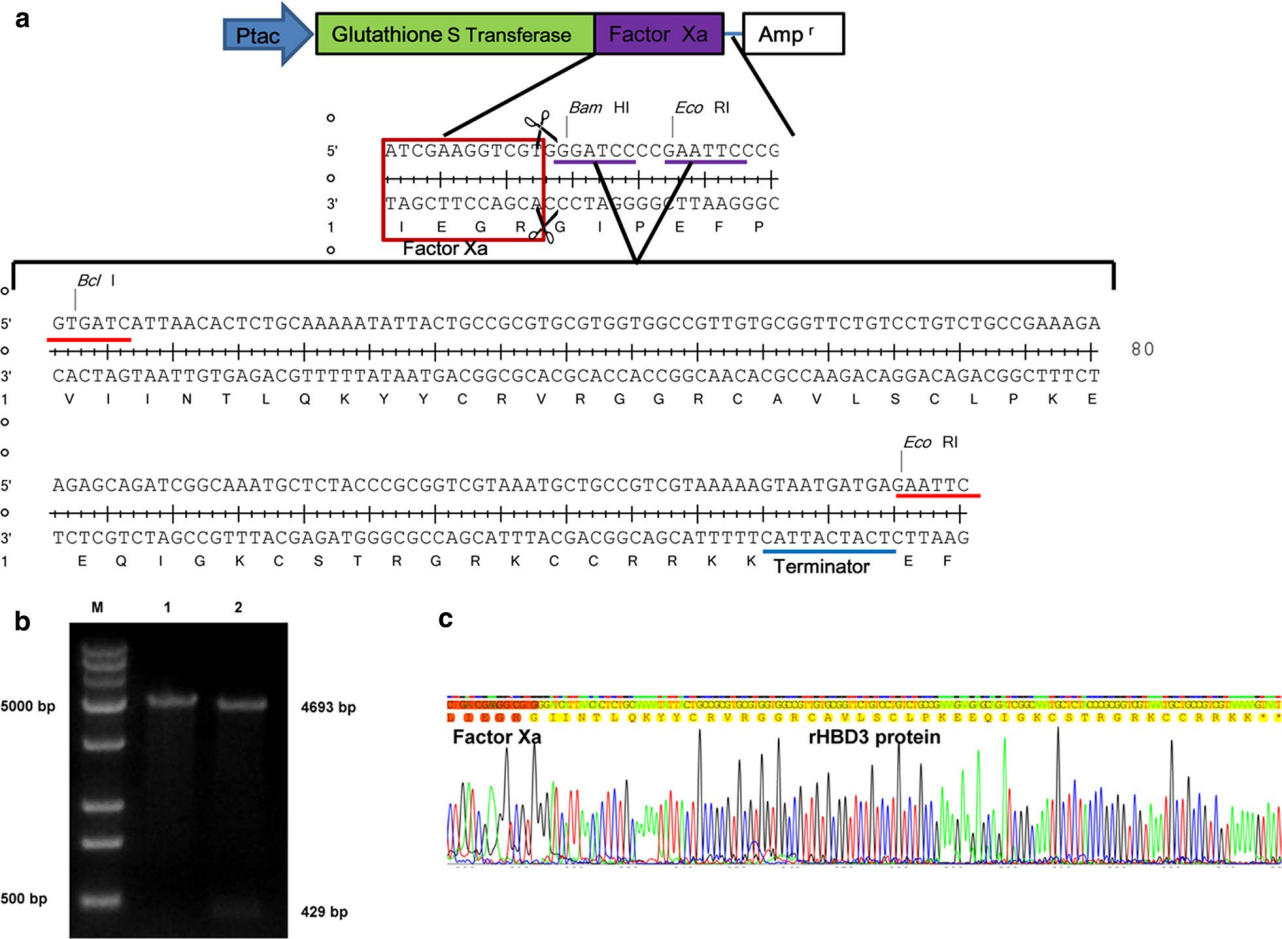
Recombinant HBD3 protein prokaryotic expression vector construction and verification. **a** Designing scheme of rHBD3 prokaryotic expression vector. **b** Restriction enzyme analysis of the PGEX-5X-HBD3 recombinant plasmid. Lane 1 is the DNA fragment recombination plasmid digested by *Eco*RI. Lane 2 is the DNA fragments of recombination plasmid digested by *Bst*BI and *Eco*RI. **c** Sequencing identification of PGEX-5X-HBD3 vector. The DNA and amino acid fragments marked by red are the *Factor Xa* recognition sites. The yellow one is the HBD3 protein sequences

The vector was designed as Fig. 1a, and the sequences were synthesized after the optimal design. The vectors and sequences were double digested by *Bam*HI, *Eco*RI and *Bcl*I, *Eco*RI separately. The recombinant vector was constructed by utilizing isocaudarner and then detected by double digestion and sequencing methods. The 1st lane in Fig. 1b is the vector digested by *Eco*RI, and the 2nd lane is the vector double digested by *BstB*I and *Eco*RI. The sequencing data revealed that the synthesized DNA fragment was correctly inserted into the vector. The amino acid fragments labeled as red is the *Factor Xa* recognition site, whereas the yellow labeled is the HBD3 protein sequence (Fig. 1c).

### Expression of recombinant HBD3 (rHBD3) protein

**Fig. 2:**
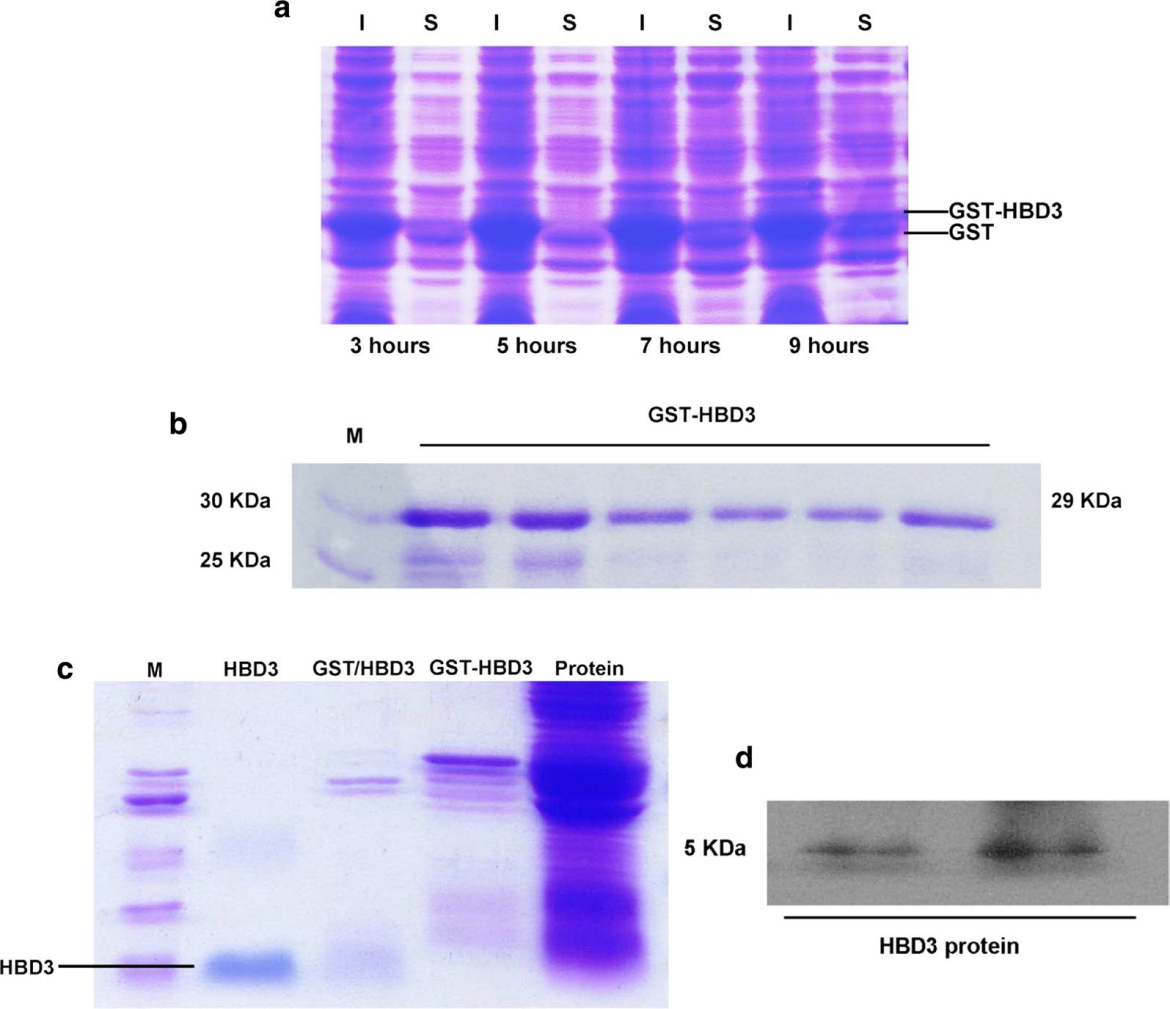
Expression and identification of rHBD3 protein. **a** Effects of different post induction times on the expression of target fusion protein with BL21 (DE3) pGEX-5X-HBD3. Soluble and insoluble proteins were analyzed at various post induction times by using SDS-PAGE (*S* soluble fractions, *I* insoluble fractions). **b** SDS-PAGE analysis of collected samples of purified fusion protein. M is the protein marker. Lanes are all the purified fusion protein. **c** SDS-PAGE analysis of protein fragment that has been digested by *Factor Xa*. M is the protein marker. Lane 1 is the purified recombination HBD3, lane 2 is the digested protein fragments lane 3 is pure GST-HBD3 protein, lane 4 is all protein. **d** Western blot analysis of recombination HBD3. Lanes are all the purified rHBD3

The optimal induce times were evaluated by SDS-PAGE. The data showed that 9 h is the optimal induction time to gain more soluble fusion HBD3 protein (Fig. 2a). Figure 2b shows that abundant fusion GST-HBD3 protein was purified by Sepharose^®^ Fast Flow system. Figure 2c reveals that GST-HBD3 protein was completely cleaved by *Xa* factors. Western blot analysis confirmed that the purified protein was HBD3 (Fig. 2d).

### rHBD3 is harmless to *Escherichia coli* (BL21)

**Fig. 3:**
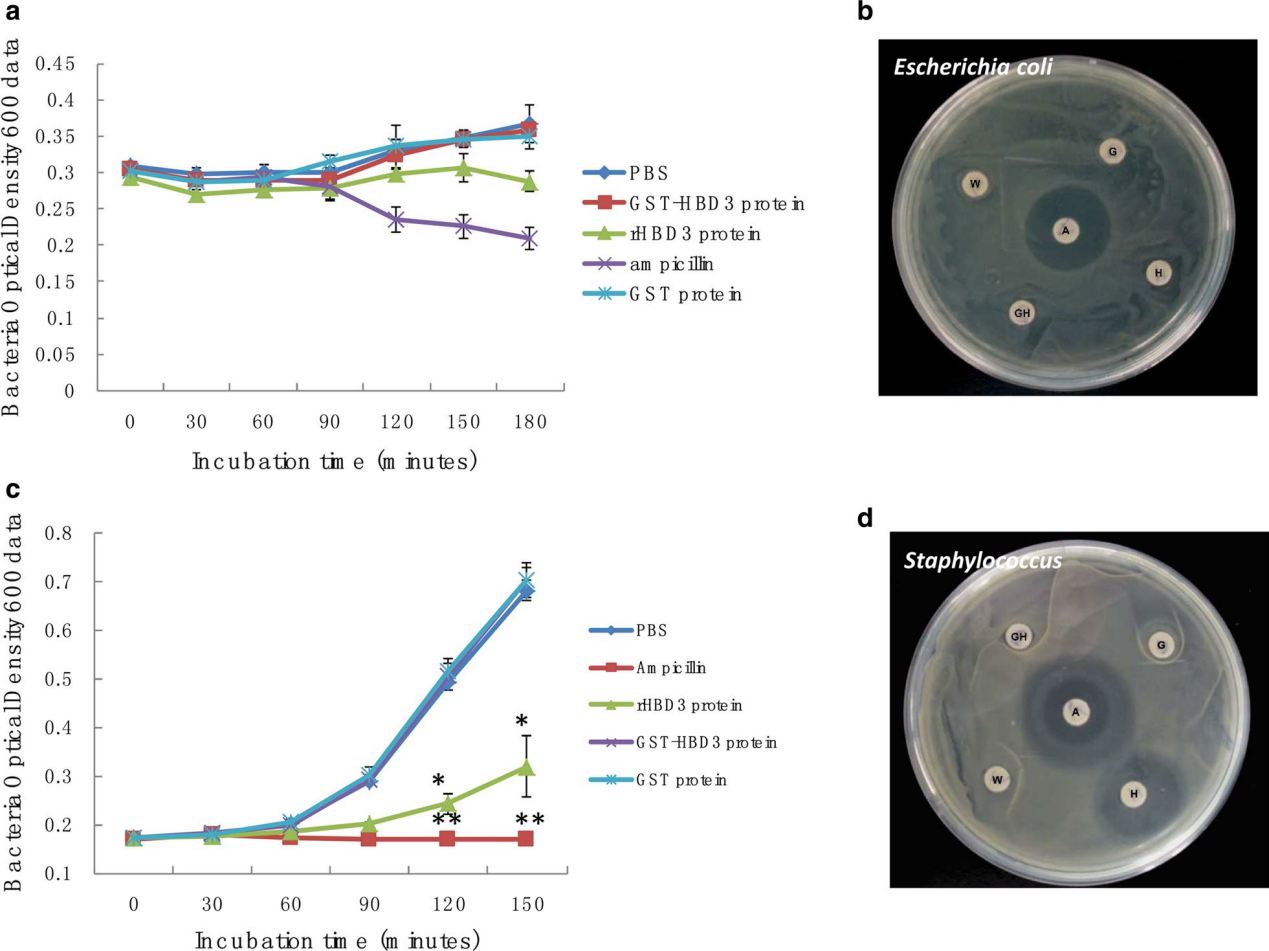
Anti-bacterial capacity analysis of rHBD3 protein by *E. coli* and *Staphylococcus*. **a** Anti-bacterial action of different proteins (GST-HBD3, HBD-3, GST, ampicillin) treated on BL21 (DE3) strain. In this experiment, the final concentration of GST tag protein, GST-HBD3 fusion protein, and rHBD3 protein was 20 μg/ml. **b** Inhibition zone of different protein (GST-HBD3, HBD-3, GST, AMP) on *E. coli* of BL21 (DE3) stain (*W* water, *G* GST tag, *GH* GST-HBD3 fusion protein, *H* recombination HBD3 protein, *A* ampicillin). **c** Anti-bacterial action of different proteins (GST-HBD3, HBD-3, GST, and AMP) treated on *Staphylococcus aureus* (ATCC 25923) strain. In this experiment, the concentration of GST tag protein, GST-HBD3 fusion protein, and rHBD3 protein was 10 μg/ml. **d** Inhibition zone of different proteins (GST-HBD3, HBD-3, GST, and AMP) on *S. aureus* (ATCC 25923) strain (*W* water, *G* GST tag, *GH* GST-HBD3 fusion protein, *H* recombination HBD3 protein, *A* ampicillin)

The anti-*E. coli* activity of HBD3 protein was evaluated by bacterial growth curve and exhibition zone tests. Results showed that either GST-HBD3 or recombinant HBD3 protein is harmful to *E. coli* grown in any concentration (Fig. 3a, b).

### rHBD3 displayed anti-*Staphylococcus* activity

The basic bioactivity of rHBD3 protein was reflected by the anti-*Staphylococcus* capacity. Results showed that rHBD3 completely inhibits the *Staphylococcus* proliferation in low concentration compared with other proteins obtained from purity experiments (Fig. 3c, d).

### rHBD3 reduced *Mycobacterial* infection capacity in alveolar epithelial cells

**Fig. 4:**
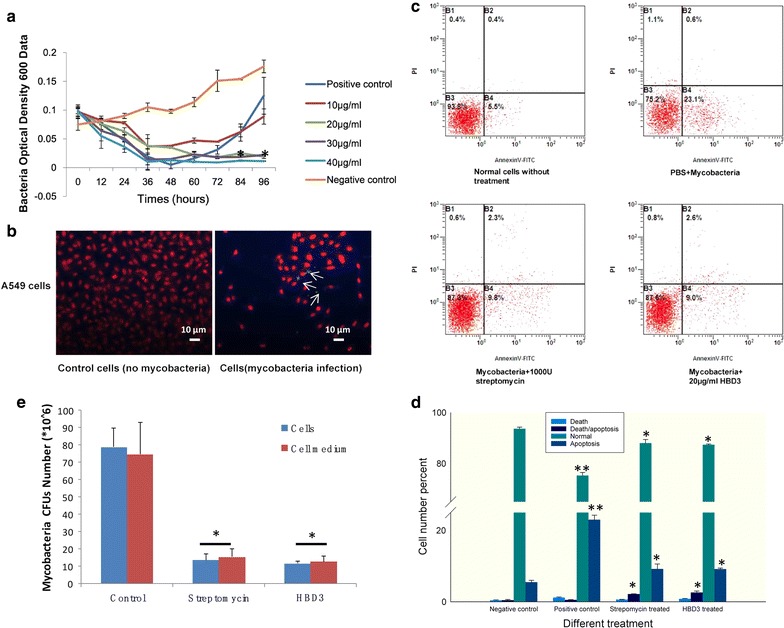
Anti-*Mycobacterium bovis* capacity of rHBD3 protein to A549 cells. **a** *M. bovis* growth curve after incubation with different concentrations of HBD-3. Negative control is *M. bovis* with Middle Brook 7H9 broth. Positive control is *M. bovis* with streptomycin (1000 U/ml). **b** *Mycobacteria* invasion tests. Yellow spots in the figure are *Mycobacteria*. **c** Cell apoptosis analysis of A549 cells infected by *M. bovis* with different treatments. **d** The data of cell apoptosis and death ratio analysis of A549 cells infected by *M. bovis* with different treatments. All the experiments were replicated three times and the changes are presented as mean ± SEM. P values < 0.05 were considered as statistically significant. **e** CFU tests in A549 cells and its medium (*P < 0.05)

The anti-bacterial capacity of rHBD3 to *M. bovis* was evaluated by its growth characteristic. Figure 4a suggests that rHBD3 protein exhibits strong anti-*M. bovis* capacity at 20 μg/ml relative to other concentration (Fig. 4a). When A549 cells were infected with *Mycobacteria* at MOI 10:1, the *M. bovis* bacteria were observed after staining with Auramine O (Fig. 4b). Subsequently, both rHBD3 protein (20 μg/ml), *M. bovis* (MOI 10:1)were mixed with A549 cells for 24 h, cells apoptosis and death ratio was evaluated by flow cytometry (FCM). The apoptosis and death ratios were evidently reduced in the rHBD3 and streptomycin-incubated group compared with those in the PBS-incubated group. However, no differences were observed in these two groups (Fig. 4c, d). *Mycobacteria* CFUs were separately detected in cells and cell medium; the results revealed that HBD3 treating *Mycobacteria* were evidently reduced both in A549 cells and its medium. However, no discrepancy was found in *streptomycin*- and HBD3-incubated group (Fig. 4e).

### rHBD3 reduced *M. bovis* infection capacity to macrophage

**Fig. 5:**
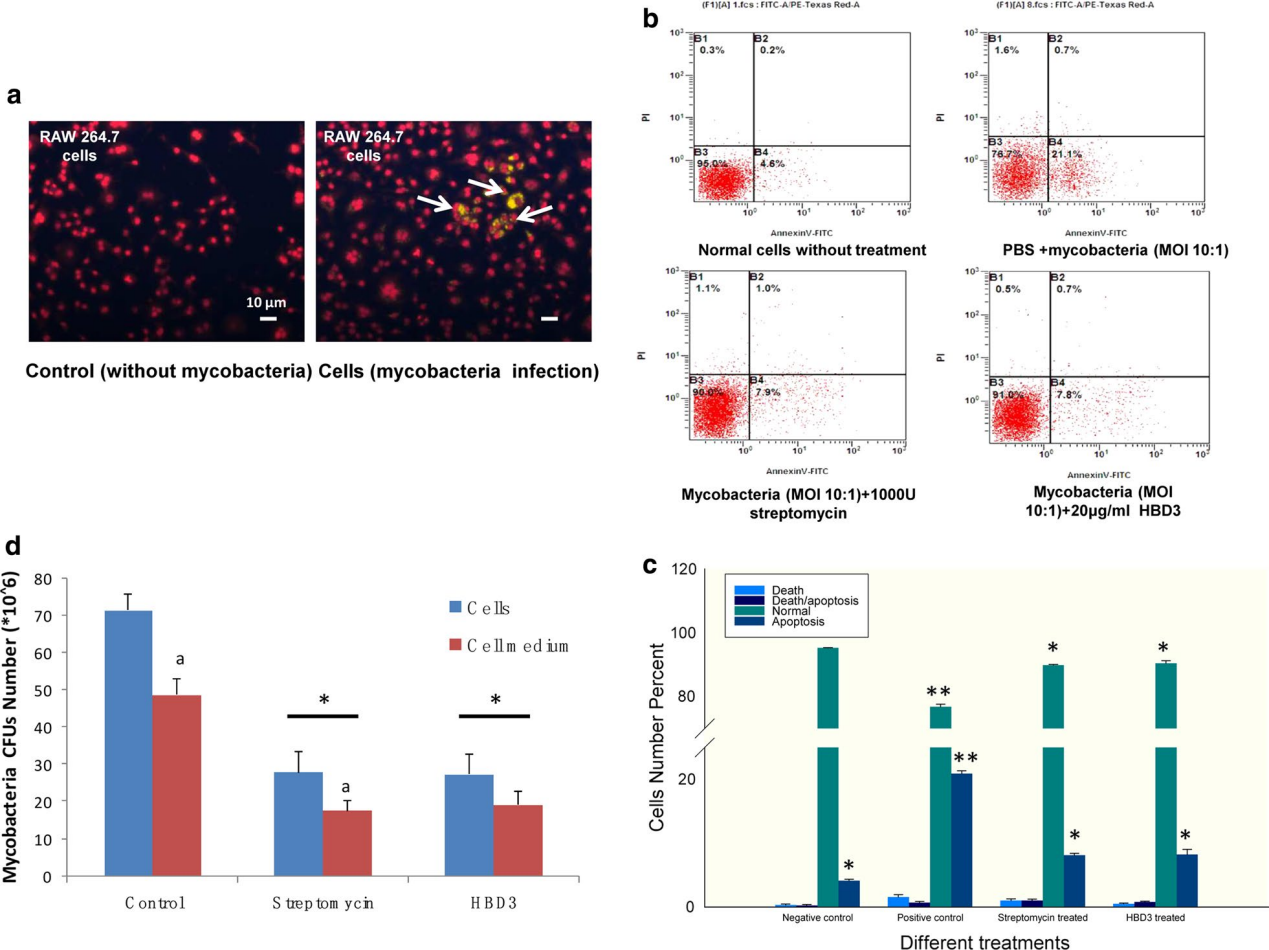
*Mycobacterium bovis* resistant capacity analysis of rHBD3 protein to macrophage. **a** *Mycobacteria* invasion tests. Yellow spots in the figure are *Mycobacteria*. **b** Cell apoptosis analysis of macrophage infected by *M. bovis* with different treatments. **c** The data of cell apoptosis and death ratio analysis of macrophages infected by *M. bovis* with different treatments. All the experiments were replicated three times and the changes are presented as mean ± SEM. P values < 0.05 were considered as statistically significant. **d** CFU tests in macrophage and its medium (a,*P < 0.05)

The anti-*M. bovis* tests were conducted in macrophage. The yellow spot in Fig. 5a revealed that *Mycobacteria* were recruited into the macrophage after dyeing with Auramine O. Subsequently, the mixture of rHBD3 (20 μg/ml) and *M. bovis* (MOI 10:1) was incubated with RAW264.7 cells for 24 h, and the cell apoptosis and death ratio was evaluated by flow cytometry (FCM). Apoptosis and death ratios were evidently reduced in rHBD3- and *streptomycin*-incubated group compared with negative control. However, no differences were observed for these two groups (Fig. 5b, c). CFU tests revealed that the *Mycobacterial* infection capacity was evidently reduced after treated with HBD3 (20 μg/ml) or streptomycin (1000 U) (Fig. 5d).

## Discussion

Human β-defensins participate in the control of *Mycobacteria* multiplication in the latent infection stage (Rivas-Santiago et al. 2006). As a star molecule of human β-defensins, HBD3 participated in the in vivo killing of pathogenic microorganism that relies on its bioactivity (Auvynet and Rosenstein 2009). A recent study reported that HBD3-transgenic cattle reduces the susceptibility to *M. bovis* infection (Su et al. 2016). However, the expression of HBD3 in vitro and its accurate inhibition concentration are not yet determined. The results of the current study suggested that purified rHBD3 is obtained by recombinant DNA technology and prokaryotic expression system. Anti-bacterial tests revealed that rHBD3 protein maintains its basic bioactivity. Anti-*Mycobacterial* capacity study showed that rHBD3 protein reduces the *M. bovis* infectivity by killing the bacteria.

Huang et al. first purified the expression of rHBD3 recombinant protein (fused with His tag) in vitro by using optimal-induced condition (Huang et al. 2006, 2007). However, the charge diversity of His tag affected the anti-bacterial property of HBD3 protein as previously reported (Carson et al. 2007; Scudiero et al. 2010). Moreover, small quantity of soluble rHBD3 protein was produced despite using optimal induction condition because of the limited water solubility of His tag. Additionally, the potential inhibiting effects of His-HBD3 protein to host bacteria existed when the protein was produced. Thus, the GST fusion expression system is the best choice for rHBD3 protein expression. Subsequently, GST-HBD3 was expressed and proved no effects to host bacteria growth (Si et al. 2007). However, the recombinant protein was not perfectly checked by western blot analysis in their study, and instead only molecular weight comparison was operated.

The optimal induction condition was evaluated to obtain more soluble protein. In this study, the rational induced time is 9 h after adding isopropyl-β-d-thiogalactopyranoside (IPTG), which is shorter than previously reported (Huang et al. 2007). The protein was digested by *Factor Xa* and purified by Sepharose^®^ Fast Flow system. *Factor Xa* digested temperature increased the degradation risk of GST-HBD3 protein relative to *TrxA* proteases (Auvynet and Rosenstein 2009). Fortunately, rHBD3 protein was not degraded for rigorous operation.

rHBD3 bioactivity was evaluated by anti-bacterial tests. The results suggested that rHBD3 protein is not harmful to *E. coli*, but the protein exhibits strong anti-bacterial activity to *Staphylococcus*, which is different from previous reports (Nuding et al. 2009). The expressed rHBD3 lost its anti-*E. coli* capacity in the current study compared with previously reported findings. One of the reason that causing this difference is the bacterial strain discrepancy, which is a result of different antibacterial spectrum. The previous study used ATCC 25922 *E. coli* strain, whereas the current study used BL21 (DE3) as a target one. The other important reason is the peptide structure differences that caused the anti-bacterial capacity discrepancy (Powers and Hancock 2003). In the current study, the peptide structure decided by GST expression system which led to antibacterial capacity discrepancy. Additionally, the anti-*Staphylococcus* activity of rHBD3 showed no obvious changes compared with that in the previous reports (Sass et al. 2010; Scudiero et al. 2010).

In a previous study, Bruno Rivas-Santiago et al. proved that β-defensins were important in early immune responses to *Mycobacterial* tuberculosis (Rivas-Santiago et al. 2005). Subsequently, they pointed out that β-defensins were expressed and play crucial roles in tuberculosis infection (Rivas-Santiago et al. 2006). The anti-*Mycobacterial* capacity of HBD3 was evaluated by H37Rv bacterial strain, and its MIC was 3.4 μM (Martinez 2008). However, its anti-*M. bovis* activity was not checked in the previous study. In the current study, rHBD3 anti-*M. bovis* capacity was evaluated by *M. bovis* growth curve and cell apoptosis tests. Results showed that rHBD3 protein suppresses the *M. bovis* multiplication as previously reported (Martinez 2008), and its minimal inhibitory concentration was lower than H37Rv strain. Cell apoptosis test revealed that the HBD3-treated *M. bovis* reduced the A549 cells and macrophage susceptibility to *M. bovis*, which is identical to the results of the previous report (Su et al. 2016). Additionally, no obvious discrepancy was observed between streptomycin- and HBD3-incubated group. Surprisingly, CFU assays exhibited that *M. bovis* counts in epithelial cells is incompletely equal to in macrophage, especially performed in cells and its medium. This discrepancy confirmed the recruitment of macrophage during *M. bovis* infection, which conforms to the result of previous report.

Auramine O was used for *M. bovis* detection; results showed that more bacteria invaded into cytoplasm, which is the same in previous reports (Alnour et al. 2012; Anthony et al. 2006). However, *M. bovis* that surrounding the cells were not counted because limiting of *M. bovis* growth characteristics. This finding exhibited that A549 cells has an important role in controlling *M. bovis* multiplication.

In summary, purified rHBD3 was obtained by recombinant DNA technology and prokaryotic expression system. Anti-bacterial tests revealed that rHBD3 protein inhibits the *Staphylococcus* multiplication rather than the host *E. coli*, which maintained its basic bioactivity. *M. bovis* inhibition test revealed that rHBD3 protein controls the *M. bovis* proliferation in 20 μg/ml concentration. In addition, *M. bovis* treated by rHBD3 protein reduced its infectivity to epithelial cells and macrophage. In conclusion, the expression of HBD3 protein inhibits *M. bovis* growth and thus is an ideal reagent for *M. bovis* prevention and therapy.

## Abbreviations

*M. bovis*: *Mycobacteria bovis*
HBD3: Human bata defensin 3
